# Magnet Ingestion in Children Management Guidelines and Prevention

**DOI:** 10.3389/fped.2021.727988

**Published:** 2021-08-04

**Authors:** Tariq Altokhais

**Affiliations:** Division of Pediatric Surgery, Department of Surgery, College of Medicine and King Saud University Medical City, King Saud University, Riyadh, Saudi Arabia

**Keywords:** magnet, ingestion, neodymium, children, toys

## Abstract

**Purpose:** Foreign body ingestion is common in children, and most foreign bodies pass spontaneously without causing serious injuries. Ingestion of multiple high-power magnet pieces is unique and increases the risk of morbidity and mortality. The longer the duration of ingestion, the increased likelihood of complications. Various management options have been reported, and there is no consensus on the ideal management which necessitates the need for a practical algorithm. The incidence of magnet ingestion has been increasing and directly related to the laws and recalls. The aim of this review is to provide an easy and practical pathway for management and to highlight the preventive rules of the legislations and recalls.

**Methods:** PubMed/MEDLINE, the Cochrane Database of Systematic Reviews, and the list of references from all identified complete publications were searched for all publications in English-language for pediatric magnet ingestion.

**Conclusion:** Practical and time-saving management pathways are recommended to minimize the risk of complications. Preventive rules and recalls are important for eliminating the availability of these hazardous magnets. Public awareness about the unique risks posed by these magnets if ingested is important.

## Introduction

Foreign body (FB) ingestion is common in children, and most foreign bodies pass spontaneously without causing serious injuries (1, 2). Various types of ingested FBs have been reported (1). However, ingestion of multiple high-power magnet pieces increases the risk of morbidity and mortality (3, 4).

In contrast to commonly ingested complicated FBs, such as batteries or sharp objects, reports of ingestion of small magnet pieces are relatively recent (1–4). Ingestion of multiple magnet pieces is peculiar, and since the first report of Neodymium magnet ingestion causing serious bowel injuries in 2002 (4), the incidence of magnet ingestion has been increasing (5–8).

Many professional health organizations recognize the risk of magnet ingestion and recommend immediate medical consultation (9). Various management options have been reported, and there is no consensus on the ideal management (7–10).

The aim of this review is to provide an easy and practical pathway for management and to highlight the incidence of magnet ingestion in relation to the legislations and recalls.

### Review and Methodology

The following sources have been searched and relevant materials have been included:

- PubMed/MEDLINE was searched for all publications in English-language journals using the following words alone or in combination:
- “pediatric,” “magnet ingestion,” “magnet injury,” “magnetic foreign body,” “magnetic toy,” “neodymium.”
- The Cochrane Database of Systematic Reviews was searched for reviews using the Medical Subject Heading: “magnet ingestion.”
- The list of references from all identified complete publications.

## Epidemiology

FB ingestion is a common problem in children regardless of their age; however, FB ingestion is more common in younger children, especially in those below 4 years of age (1).

Since the first report of magnet ingestion by McCormick et al. in 2002 (4), many reports describing the clinical picture, complications, and management have been published (5–10).

Many professional health organizations have recognized the dangers of magnet ingestion and reported their incidence (9). The true incidence is probably under-reported due to the number of magnet pieces that passed without causing clinical problems (8, 10). However, there is a clear increase in the incidence from 2000 to 2020. The National Electronic Injury Surveillance System estimated that there were 16,386 possible magnet ingestions among children aged <18 years from 2002 to 2011 in the United States (8). The numbers have risen steadily and fluctuated corresponding to time periods during which federal laws and court decisions were on or off the market (9–13).

In the past, many reports addressed the hazards and potential risks of disk battery ingestion, but hazardous materials are increasing as the industry produces new materials (11). Moreover, sharp metallic darts used in target games have been abandoned from the market due to the risk of serious injuries and replaced by magnetic darts (11). Many children's toys use tiny magnetic pieces that become a problem when ingested, and the other materials which may be produced by the industry and cause serious injuries need to be considered.

## Neodymium vs. Conventional Magnet

Rare earth metals, such as neodymium, are highly powered magnets commonly used in industry, mainly because of their impressive strength-to-size ratio. The bounding strength is 5–30 times that of a conventional magnet (5–9). General Motors and Sumitomo Special Metals (Japan) invented these special magnets in 1982, and these have been widely used in toys, kitchen utensils, desk items, and many household products (7–13).

Magnet pieces attract each other across the walls of the gastrointestinal tract, causing ischemia, tissue necrosis, perforation, fistula formation, obstruction, peritonitis, or death (5–15).

## Clinical Presentation

**Figure 1:**
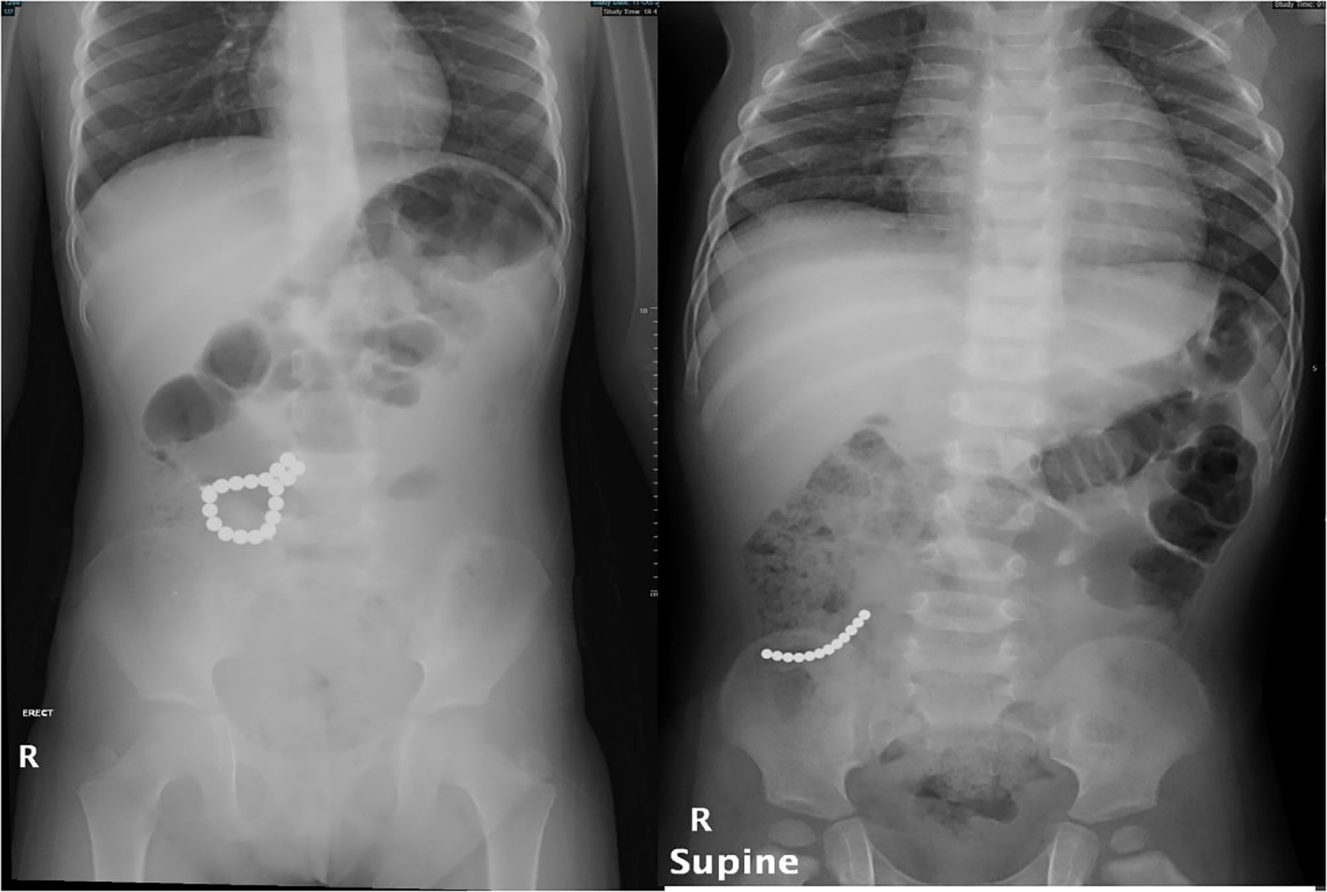
Abdominal radiograph of two patients who presented with non-specific abdominal symptoms.

Most children are asymptomatic in the early phase of ingestion. Symptoms progress according to the duration of ingestion and the location of the magnet. Symptoms are usually non-specific; however, vomiting and abdominal pain are the two most common symptoms (6–8). Most patients who present with vague abdominal pain are diagnosed with an abdominal radiograph (Figure 1).

**Figure 2:**
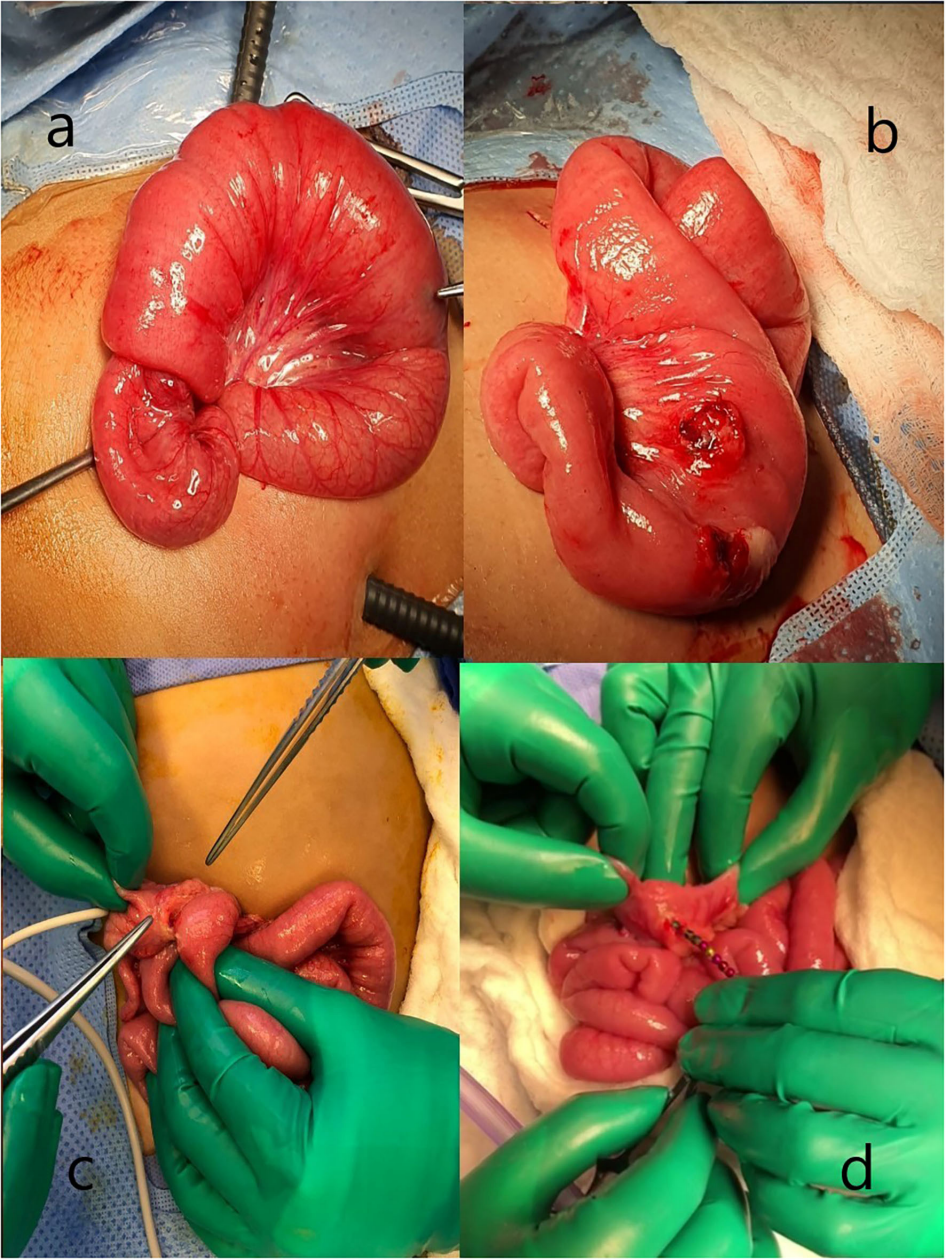
Operative pictures of complications. **(a,b)** Ileo-ileal intussusception because of magnet ingestion. **(c,d)** Ileocolic fistula caused by magnet pieces.

Delay in treatment results in more complications, including fistula formation (enteroenteric, arteriogastrointestinal, or tracheoesophageal), ulceration, perforation, stricture, obstruction, hemorrhage, mediastinitis, peritonitis, volvulus, sepsis, and death (8, 16–23) (Figure 2). The first report of death following magnet ingestion was published in 2006 in a 20-month-old child (24). Since then, many case reports followed, and legislations and laws were established for the abandonment of magnet use (25–37).

## Management

Ingested FBs are generally managed by different specialties, such as pediatric surgery, pediatric ENT, general pediatrics, and pediatric gastroenterology. These different specialties reported different management options and outcomes, which could be explained by the different clinical features of patients in each specialty (10). In contrast, the ingestion of magnets is different. The number of pieces and the ingestion interval between magnet pieces should be determined; the longer the interval period, the greater the risk of complications (16, 17).

We have managed many cases of magnet ingestion, either single or multiple. Eventually, most of our patients needed intervention for removal either by endoscopy or surgery. We counted the number of pieces and correlated it with the number in the X-ray scan; if there was a discrepancy, we performed an intraoperative X-ray scan to confirm the removal of all pieces. Among the many patients who ingested multiple pieces, only two patients were managed conservatively by observation. These patients swallowed the magnet pieces together, with no interval between swallowing. All patients who swallowed a single piece were observed, and all of them passed the magnet piece in <24 h.

**Figure 3:**
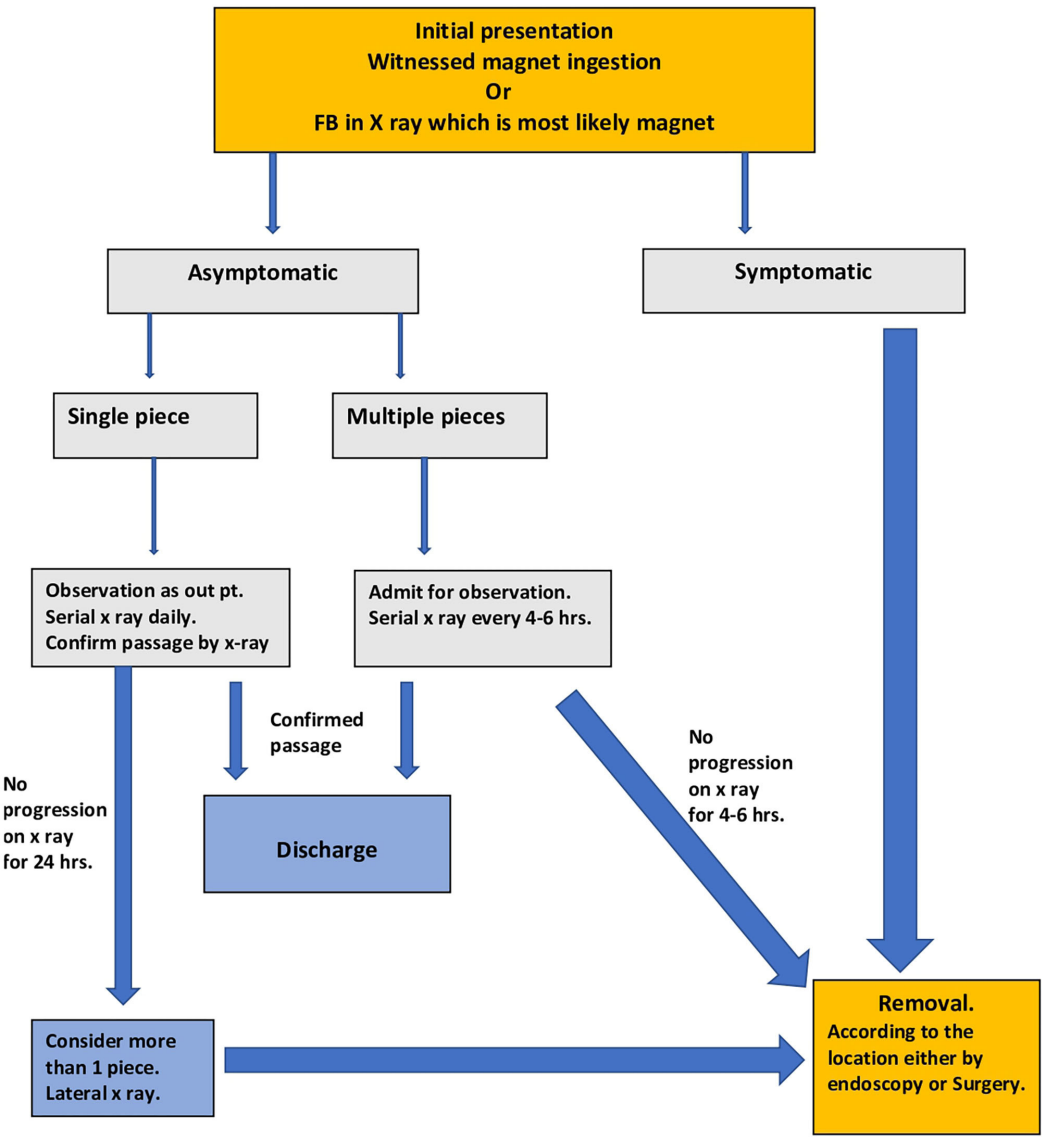
Management pathway for magnet ingestion.

The management of ingested magnet pieces is completely different from the management of other FB ingestions. Hussain et al. proposed the first algorithm for the management of ingested magnets, and the algorithm was adopted by the North American Society for Pediatric Gastroenterology, Hepatology, and Nutrition (NASPGHAN) in 2015 (5, 38). The algorithm is detailed, has many treatment options, and advocates prolonged observation. However, the increased number of reported cases and complications due to prolonged observation necessitates the need for a practical and easy algorithm, as proposed in (Figure 3). The algorithm is self-explanatory, and the duration of ingestion is crucial for decision-making. The observation period and the decision for removal are based on the normal physiologic small bowel transit time and should not exceed 6 h (39, 40).

If magnet ingestion was witnessed or if magnet pieces were seen in the abdominal X-ray scan and the child is symptomatic, a decision is made for immediate removal. Removal can be achieved by endoscopy if the magnet pieces are in the esophagus, stomach, duodenum, or colon. A surgical consultation is warranted in cases where endoscopic removal cannot be achieved or if a complication occurs during retrieval. Surgical removal can be performed either by laparotomy or laparoscopy, according to the facility and experience. Laparoscopic removal might be challenging owing to the magnets adhering to the instruments.

The number of magnet pieces—two or more—must be determined if the child is asymptomatic. If there is a single magnet, without symptoms, the child can be treated as an outpatient with daily X-ray scans. If the child passes the magnet, a confirmatory X-ray scan should be performed. If the magnet piece does not progress in a daily X-ray scan, possibility of more than one piece should be considered, especially if these two pieces are identical. A lateral X-ray scan should be performed, although, it is sometimes impossible to determine the number of pieces. If the single piece does not progress in 24 h, removal should be attempted according to the location.

For asymptomatic multiple magnet pieces, serial X-ray scans should be performed every 4–6 h. If the pieces progress, a confirmatory X-ray scan should be performed to ensure that it has passed through the rectum. If the pieces do not progress in 6 h, removal should be considered without delay. Delaying removal might cause complications such as fistula formation or perforation.

### Single vs. Multiple Magnets

On rare occasions, it is confusing to distinguish single from multiple magnets in the plain X-ray scan, and a misdiagnosis results from a false assumption that a single magnet is present (1). Radiological methods may not detect whether the identified objects are magnetic, single, multiple, or multiple pieces bound together, or if they are in different intestinal tract locations (3, 17). Both plain X-ray and computed tomography scans lack sensitivity to determine the multiplicity of magnet pieces (1). A single bead-like magnet piece appears as multiple and two identical bound pieces look like a single piece in the X-ray scan. Thus, failure of passage of a single piece after 24 h of observation should be treated as multiple pieces.

## Prevention and Relation to Legislation

The death of a 2-year-old boy in Washington State, after swallowing magnet pieces (Magnetix, Rose Art Industries Inc., Livingston, NJ, USA), was the first event leading to the recall of the magnet set by the US Consumer Product Safety Commission (CPSC) (1).

There is a strong relationship between the incidence of magnetic ingestion and legislative laws. For instance, in the United States of America (USA), the CPSC halted the sale of high-powered magnet sets and finalized a federal rule in 2014. Subsequently, there was a significant reduction in the number of patients. However, the recall was overturned by the Federal Court for the District of Colorado in 2018, and the magnet sets could be sold to anyone over 14 years of age. Since then, there has been a 444% increase in magnet-related calls to the Poison Control Center (9, 30–34).

We noticed a huge number of cases of magnet ingestion in Saudi Arabia until the magnet sets were banned from the market by the Defective Products Recall Center in February 2020 (Recall reference number: 20020-20023)^1^. The number of cases decreased dramatically at the local and national levels after the ban. However, a year after the ban, the number of cases steadily increased. This could be explained by the unnoticed resale of these magnets.

In addition to the restriction of manufacturing of rare earth magnetic toys, parents and families should be educated as much as possible through schools' educational programs, social media, and public media channels, about the unique risks posed by these magnets if ingested.

## Conclusion

The incidence of magnet ingestion in children is directly related to their availability and the legislation concerning the magnets. Preventive rules and recalls are important for eliminating the availability of these hazardous magnets. Practical and time-saving management pathways are recommended.

## Author Contributions

The author confirms being the sole contributor of this work and has approved it for publication.

## Conflict of Interest

The author declares that the research was conducted in the absence of any commercial or financial relationships that could be construed as a potential conflict of interest.

## Publisher's Note

All claims expressed in this article are solely those of the authors and do not necessarily represent those of their affiliated organizations, or those of the publisher, the editors and the reviewers. Any product that may be evaluated in this article, or claim that may be made by its manufacturer, is not guaranteed or endorsed by the publisher.

## Footnotes

^1^ https://recalls.sa/Recall/Search?TradeMarkTypeId=&TradeMarkId=&ModelId=&ReferenceNo=20022&RecallDateFrom=&RecallDateTo=
